# A case of osmotic demyelination syndrome occurred after the correction of severe hyponatraemia in hyperemesis gravidarum

**DOI:** 10.1186/1472-6823-14-34

**Published:** 2014-04-11

**Authors:** Giovanni Corona, Luigi Simonetti, Corinna Giuliani, Alessandra Sforza, Alessandro Peri

**Affiliations:** 1 Medical Department, Endocrinology Unit, Azienda USL Bologna, Maggiore-Bellaria Hospital, Bologna, Italy; 2 Emergency Interventional Radiology and Neuroradiology Unit, Azienda USL Bologna, Maggiore-Bellaria Hospital, Bologna, Italy; 3 Department of Experimental and Clinical Biomedical Sciences, Endocrine Unit, University of Florence, Florence, Italy

**Keywords:** Osmotic demyelination syndrome, Hyponatraemia, Pregnancy

## Abstract

**Background:**

Osmotic demyelination syndrome (ODS) may be observed as a result of a rapid change in serum osmolarity, such as that induced by an overly rapid correction of serum sodium levels in hyponatraemic patients.

**Case presentation:**

We describe the case of a 21-year-old woman who was hospitalized at week 10 of gestation because of severe hyperemesis. At admission the patient appeared restless and confused and severe hyponatraemia (serum sodium 107 mmol/L) and hypokalemia (serum potassium 1.1 mmol/L) were detected. Active and simultaneous correction of these imbalances led to an overly rapid increase of serum sodium levels (17 mmol/L in the first 24 hours). Isotonic saline solution was stopped and replaced by 5% dextrose solution infusion. However, the neurological alterations worsened and the radiological features were consistent with the diagnosis of extra-pontine ODS. Steroids were administered intravenously with progressive improvement of biochemical and clinical abnormalities. At the time of discharge, 20 days later, the patient was able to walk and eat autonomously with only minimal external support.

**Conclusions:**

This report illustrates an unusual case of ODS, occurred after an excessive rate of correction of hyponatraemia obtained with isotonic saline infusion. Hypokaliemia and its active correction very likely played a crucial role in facilitating the onset of ODS. This interesting aspect will be explained in detail in the article. A more cautious and thoughtful correction of electrolyte alterations, would have probably prevented the onset of ODS in this patient. Physicians should be aware of the possibly fatal consequences that an exceedingly rapid change of serum osmolarity may have and should strictly follow the known safety measures in order to prevent it to occur.

## Background

Osmotic demyelination syndrome (ODS) is an uncommon disorder, characterized by non-inflammatory demyelination involving the pons and other areas of the central nervous system, which may occur for instance as a consequence of an overly rapid correction of hyponatraemia [1]. Here we report a case of ODS occurred after correction of hyponatraemia with isotonic saline solution in a pregnant woman with hyperemesis gravidarum and concomitant other electrolyte disorders, which are additional risk factors for the development of this syndrome. Some criticisms in the management of the patient, which undoubtedly eased the occurrence of ODS, will be highlighted.

## Case presentation

**Figure 1:**
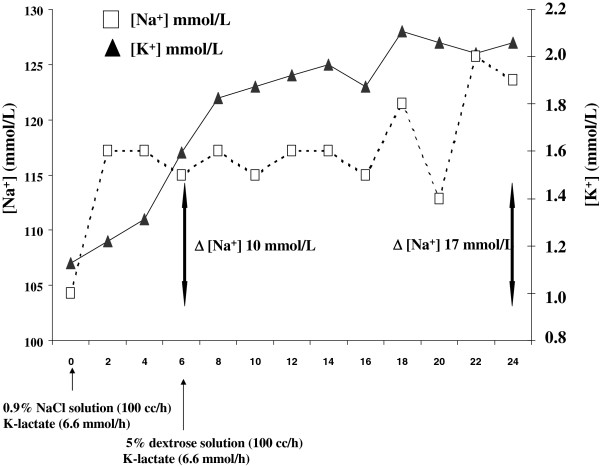
**Serum [Na**^**+**^**] and [K**^**+**^**] progression during active treatment for the correction of severe hyponatraemia and hypokalemia in the patient.**

**Figure 2:**
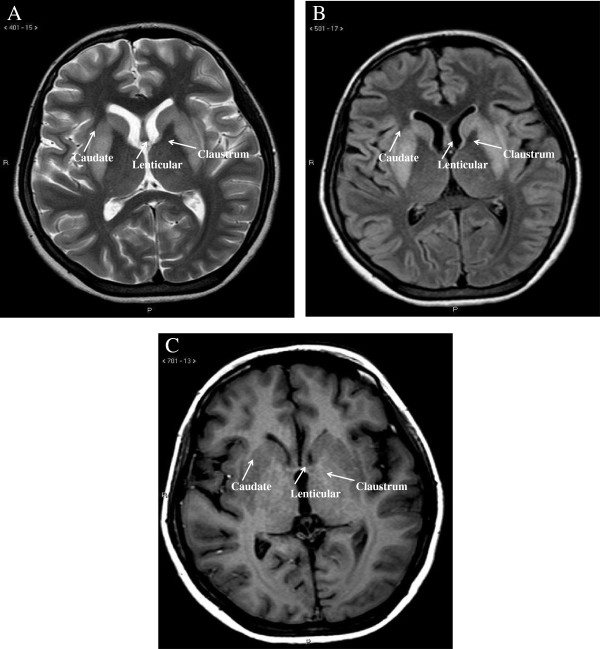
**MRI features.** Signal intensity of the lenticular, claustrum, and caudate cerebral nuclei on axial T2 weighted images **(A)**, FLAIR images **(B)** and Tl weighted images **(C)**.

Hyperemesis gravidarum is a condition characterized by severe vomiting resulting in dehydration, fluid electrolyte imbalance and weight loss [2]. We report the case of a 21-year-old woman who was hospitalized at week 10 of gestation due to severe hyperemesis. The condition appeared at week 4 of pregnancy and caused her weight to drop from 51 kg to 46 kg. The day of admission to the hospital she became restless and confused. Clinical examination performed in the emergency room revealed the presence of mild dehydration (dry mouth and skin) with normal blood pressure, increased heart rate and slightly reduced breath frequency (Table 1). Biochemical assessment performed at admission revealed severe hyponatraemia (107 mmol/L) and hypokalemia (1.1 mmol/L) (Table 1 and Figure 1) with secondary metabolic alkalosis and partially compensated respiratory acidosis (Table 1). Urinary data suggested the presence of extra-renal loss of Na^+^ and K^+^ with appropriate increase of urinary water and electrolyte retention (Table 1). The patient was promptly admitted to the intensive care unit of the hospital and intravenous isotonic NaCl solution along with K-lactate supplementation was started (Figure 1). The electrolyte disorder was intensively monitored with blood sample evaluation performed every two hours. However, during the first six hours after admission an excessive rate of correction of serum Na^+^ concentration ([Na^+^]) occurred (Δ 10 mmol/L). Hence, isotonic solution was replaced with hypotonic solution (5% dextrose, Figure 1); K-lactate supplementation was maintained. However, after 24 hours serum [Na^+^] was 124 nmol/L (Δ 17 mmol//L) and at 48 hours 128 nmol/L. The neurological symptoms worsened with the appearance of hypotonia, tremors and involuntary muscle spasms. Hormonal data showed the presence of thyrotoxicosis likely induced by hCG; serum ACTH and cortisol levels showed no abnormalities. In addition, laboratory data (e.g. reduced total proteins, albuminemia, retinol binding protein, creatinine, azotemia) indicated the presence of relatively recent, important malnutrition (Table 1). Electroencephalogram showed a non-specific slow wave activity consistent with the presence of a metabolic disorder. Magnetic resonance imaging (MRI) of the brain revealed, despite a normal pons, bilateral increased signal intensity of the lenticular, claustrum, and caudate cerebral nuclei on axial T2 weighted images (Figure 2, panel A) and FLAIR images (Figure 2, panel B). In addition, the Tl weighted images showed moderate hypointensity in the same nuclei (Figure 2, panel C). These foci did not enhance with GDTPA. The clinical and radiological picture was considered to be consistent with extra-pontine ODS associated with an overly rapid correction of hyponatraemia, which was caused by hyperemesis gravidarum At that point, intravenous steroid infusion and enteral nutrition support were started with slow improvement of biochemical and clinical abnormalities. The gynecological examination and ultrasound fetal monitoring revealed no alterations and were in agreement with the gestational age. The patient and her husband were informed on the possible fetal neurological risks and decided to continue the pregnancy. At the time of discharge, 20 days later, the patient was able to walk and eat autonomously with only minimal external support; serum electrolytes and nutritional parameters were normal. The patient delivered a healthy male at 36 weeks of gestation (caesarean section). At 4 months of age the child is apparently healthy. Unfortunately, only a mild improvement of the neurological alterations of the mother has been obtained despite physical therapy.

**Table 1:** Clinical and biochemical features of the patient

**Clinical data**		
Weight (kg)	46	
Height (cm)	160	
BMI (Kg/m^2^)	17.9	
SBP (mmHg)	120	
DBP (mmHg)	60	
HR (bpm)	120	
RF (bpm)	10	
**Laboratory parameters**		
**Serum data**	**Value**	**Normal range**
Glycaemia (mmol/L)	4.17	(3.3-6.5)
Na^+^ (mmol/L)	107	(135-146)
K^+^ (mmol/L)	1.1	(3.5-5.0)
Ca^++^ (mmol/L)	2.5	(2.18-2.58)
Mg^++^ (mmol/L)	0.90	(1.02-1.84)
Phosphate (mmol/L)	0.41	(0.48-1.45)
Creatinine (μmol/L)	36.24	(50-90)
Urea (mmol/L)	1.83	(2.5-8)
Total protein (g/L)	52	(60-80)
Albumin (g/L)	30	(35-50)
Prealbumin (mg/L)	127	(200-400)
Osmolarity (mmol/kg)	254	(275-295)
Retinol binding protein (mg/dl)	1.33	(1.55-4.59)
TSH (mlU/L)	0.01	(0.4-5.0)
fT3 (pmol/L)	7.06	(3.5-6.5)
fT4 (pmol/L)	28.31	(9-21.80)
ACTH (pmol/L)	2.26	(1.3-16.7)
Cortisol (nmol/L)	606.98	(110-607)
**Urinary data**		
Na^+^ (mmol/L)	3	
K^+^ (mmol/L)	2	
Osmolarity (mmol/kg)	418	(500-1500)
**Hemogasanalysis data**		
pH	7.65	
pO_2_ mmHg	69	
pCO_2_ mmHg	58	
BE mmol/L	37.4	
HCO_3_^-^ mmol/L	63.9	
AG mmol/L	-10	

*BMI* = body mass index, *SBP* = systolic blood pressure, *DBR* = diastolic blood pressure, *HR* = heart rate, *RF* = respiratory frequence, *bpm* = beats per minute, *BE* = base excess, *AG* = anion gap.

The case presented here describes a condition of ODS associated with overly rapid correction of serum [Na^+^] in a patient with severe hyponatraemia. ODS was first described in 1959 in a series of alcoholic and malnourished patients who developed quadriparesis and pseudobulbar palsy. Post-mortem assessment revealed the presence of a demyelinative lesion within the central pons [3]. Its relationship with electrolyte disorders was not apparent, because routine measurement of serum electrolytes was not available at that time. A link between ODS and overly rapid correction of hyponatraemia was established only in the mid-1970s [4]. ODS is recognized as a rather rare disease, although the exact frequency with which occurs is not known. The largest autopsy series indicated a prevalence of 0.25-0.5% in the general population with a peak incidence in males aged 30 to 60 years [1]. From a pathological point of view, ODS is characterized by loss of myelin sheath, whereas axons and neurons are relatively spared [5]. Traditionally, ODS has been associated with rapid correction of hyponatraemia; however, a number of different conditions have been reported in association with the development of this syndrome, independent of serum [Na^+^] alterations. The most common predisposing condition is chronic alcoholism, which was not present in our case [6]. Many patients developed ODS during the terminal stage of binge drinking; however, a few case reports of ODS after alcohol withdrawal have been reported [7]. In these instances, the osmotic change due to a decreased intake of food or water that occurs during binge drinking or alcohol withdrawal may be the cause of ODS development.

ODS has been described in patients after liver transplant, after treatment for hyperammonemic disease or with dialysis disequilibrium syndrome. This syndrome is characterized by the occurrence of neurological signs and symptoms attributed to cerebral edema during or following shortly after intermittent hemodialysis and is associated particularly with “aggressive” (high solute removal) dialysis [8,9]. The occurrence of ODS has been also associated with hyperglycemia or with the correction of hyperglycemia [10,11]. Severely burned patients are especially susceptible to ODS, which is tightly associated with serum hyperosmolality resulting from hyperglycemia, azotemia and/or hypernatraemia in these patients [12]. In pregnant women, ODS may occur following rapid correction of hyponatraemia, which may be observed in hyperemesis gravidarum [2], as in the case described here. Furthermore, several cases of ODS in women with post-partum hypernatraemia have been described in India [13]. The authors suggested that a possible cause of hypernatraemia in these patients might be the presence of gestational diabetes insipidus together with the habit to restrict fluid intake during puerperal state in some regions of India.

Overall, these findings strongly suggest that any change in brain cellular volume as the result of a rapid change in serum osmolarity may cause an injury to myelin sheats. In chronic, but not in acute, hypoosmolar states brain cells adapt by decreasing their intracellular solute content. Thus, the risk to develop ODS is substantially associated with chronic hyponatraemia. Noticeably, glial cells have a critical role in brain water handling. These cells, and not neurons, swell after hypotonic stress, thus suggesting the existence of glia-specific water pores (i.e. aquaporin 1 and 4) [14]. Brain adaptation to cellular swelling requires 24-48 hours and is driven by glial cells, which expel solutes followed by water. This is an energy-dependent phenomenon and requires the activity of the Na^+^-K^+^-ATPase pump. If a rapid correction of a hypoosmolar state then occurs, glial cells start to increase the production of organic osmolites and the intracellular ion content, in order to reverse the previous modification and regulate cellular volume [15]. However, the synthesis of organic osmolites, as well as the up-regulation of ion pumps, require time and causes energy deprivation. Thus, if an overly rapid correction of a hypotonic state occurs, the cells may not be able to effectively respond to osmotic changes and water moves into the extra-cellular space [15]. Consequently, the resulting shrinkage of glial cells may cause axonal shear damage. Oligodendrocyte apoptosis may also be induced [15], which may lead to disruption of the blood-brain barrier. The latter may cause inflammatory demyelination and further oligodendrocyte damage [15,16].

Besides serum sodium imbalance, other electrolyte alterations, such as hypophosphataemia and hypokalemia, which were also present in our case, have been associated with the development of ODS. Acute hypophosphataemia is a recognized complication of refeeding syndrome when mineral supplementation is inadequate. The phosphate depletion causes failure to convert ADP into ATP leading to an intracellular energy crisis [17], which likely causes altered function of the Na^+^-K^+^-ATPase pump and consequently brain cells dysfunction. In addition, because phospholipids are a major component of myelin, phosphate depletion may disrupt myelin integrity [18]. With regard to hypokalemia, the mechanism leading to an increased risk of ODS is not completely clear. Nevertheless, different possibilities have been proposed [1]. For instance, a reasonable explanation is that when hyponatraemia is also present, which was the situation of the case reported here, its correction rate may be increased by the simultaneous correction of hypokalemia. This statement is based on the observation that the Na^+^-K^+^-ATPase pump on the cell membrane expels Na^+^ as K^+^ enter the cell to replenish depleted intracellular stores. The consequence is a more rapid rate of correction of serum [Na^+^], which increases the risk to develop ODS. The simultaneous correction of hypokaliemia may has been therefore an important trigger for the overly rapid correction of hyponatraemia observed in this case and for the further raise occurred also after the replacement of saline infusion with 5% dextrose solution. With this critical aspect in mind, admittedly a more cautious correction of hyponatraemia and a more appropriate intervention aiming to re-lower serum [Na^+^], i.e. using also desmopressin, would have likely prevent the onset of ODS.

Clinically, ODS may present with a variety of neurological signs and symptoms, depending on the severity of the involvement and on the affected areas. In about 50% of cases isolated central pontine myelinolysis (CPM) occurs, whereas the remaining cases are more or less equally divided into isolated extra-pontine myelinolysis (EPM) or a combination of CPM and EPM, with a slight prevalence of the latter condition [19]. A number of sites may be involved, besides the pons, and among the most frequently affected sites there are the cerebellum, lateral geniculate bodies, external and extreme capsule and hippocampus. A complete list of affected areas is shown in Table 2 and includes caudate and lenticular nuclei and the claustrum, three extra-pontine areas in which lesions were observed in the patient described here. In CPM usually the clinical presentation includes dysarthria and dysphagia secondary to the involvement of corticobulbar fibres, flaccid quadriparesis followed by spasticity, due to corticospinal tract involvement, oculomotor abnormalities. Changes in mental status and conscious level may occur. In EPM a variety of behavioral changes and movement disorders may be present. These include parkinsonism, dystonia, mutism, catatonia and dysphonia [19]. Usually, these alterations develop several days after the correction of the hypoosmolar condition, although encephalopathy may be already present after a few hours [6]. A possible case of ODS with peripheral nervous system involvement after hyponatraemia correction, causing bilateral and symmetrical motor demyelinating polyneuropathy in the lower and upper extremities in association with central nervous system involvement, has been described [20]. However, admittedly systematic neurophysiological screening of an adequate series of patients with ODS would be required in order to confirm this hypothesis.

**Table 2:** Cerebral areas involved in CPM and EPM

**Common**	**Rare**
Pons	Internal capsula
Cerebellum	Claustrum
Lateral geniculate body	Midbrain
Extreme capsule	Mammillary body
External capsule	Medulla oblongata
Putamen	Internal medullary lamella
Hippocampus	
Thalamus	
Cerebral cortex/subcortex	
Caudate nucleus	

The diagnosis of ODS is based initially on clinical suspicion. Any patient with new onset neurological signs/symptoms and with a recent rise in serum [Na^+^] and/or with associated risk factors for ODS, as mentioned previously, should be considered possibly affected by ODS. Clinical findings have to be correlated with radiological assessment. MRI of the brain is the gold standard imaging technique to reveal ODS lesions. These include hypointense T1-weighted lesions and hyperintense lesions demonstrated on T2-weighted and FLAIR images. The lesions are non-contrast enhancing. All these features are in agreement with the findings we observed in our case. The time of appearance may vary and may be delayed; sometimes lesions appeared at MRI even 2 weeks after the onset of clinical alterations [21]. Typically, radiological findings do not improve over time, despite a complete clinical recovery [22].

The prognosis of ODS has been considered very negative for a long time and before the availability of modern imaging techniques usually ODS was a post-mortem diagnosis. The first in-life diagnosis was formulated only 10 years after the first description of ODS. In recent years, the mortality rate has markedly decreased and patients with complete recovery are not rarely observed. In a series of 44 German patients with ODS, for instance, only 6% died during the observation period and 40% recovered without apparent neurological alterations [22].

With regard to treatment, the best therapy for ODS would be…to prevent it to occur! Particular attention should be dedicated to patients presenting conditions known to be associated with a higher risk to develop this condition. As a general rule, the rate of correction should not exceed 12 mmol/L within 24 hours, or 18 mmol/L within 48 hours. These limits should be reduced to 8 mmol/L within 24 hours and 12 mmol/L within 48 hours in patients with chronic hyponatraemia and associated risk factors for developing ODS, which include very low serum [Na^+^], hypokalemia, malnutrition and hypophosphataemia, which were present in the case reported here [23,24]. In terms of more prudential approaches, it has also to be considered, as reported by the recently published “expert panel recommendations” on hyponatraemia, that a 4-6 mmol/L increase in serum [Na^+^] is already sufficient to reverse the most serious manifestations of acute hyponatraemia [24]. In this patient simultaneous correction of severe hyponatraemia and hypokalemia resulted in a 24-hour increase of serum [Na^+^] of 17 mmol/L. In such instances, uncertainty regarding the optimal approach still exists, mostly due to the lack of trials in human subjects. Re-induction of hyponatraemia by using desmopressin, intravenous 5% dextrose solution or a combination of both is usually unnecessary in acute hyponatraemia, may be optional in patients with low to moderate risk of ODS, but it is definitively recommended in patients at high risk of ODS [24]. The occasional use of corticosteroids has been reported in the literature. A possible favorable effect of corticosteroids in ODS, which were administered to the patient described here, is to stabilize the blood-brain barrier [1], although there is no definitive evidence of a clear benefit in humans. Urea or myoinositol have been used in animal studies, aiming to restore intracellular organic osmolites, thus protecting from cellular dehydration [1]. Prompt treatment with minocycline, a selective and potent inhibitor of microglial activation, has been proven to be effective in counteracting the accumulation and the pro-inflammatory activity of microglia, which usually occurs in demyelinative lesions in ODS [25]. Plasmapheresis has been used in a few patients and its beneficial effect has been possibly related to the reduction of inflammatory mediators and consequently to a protective effect on the blood-brain barrier [1]. Similarly, a few cases of successful treatment with intravenous immunoglobulin have been reported [26]. The mechanism of action of immunoglobulin in reversing the effects of demyelination remains to be elucidated, but it includes the possible reduction of myelinotoxic substances and the induction of remyelination [27]. Dopaminergic molecules appear helpful in improving neurological alterations once ODS is fully established [5]. Besides pharmacological treatment, the approach to ODS includes physical therapy, similarly to other neurological disorders.

## Conclusions

We reported here a case of ODS occurred in a pregnant woman with hyperemesis gravidarum after an overly rapid correction of low serum [Na^+^] in the presence of several concomitant risk factors. The overly rapid increase of serum sodium may has been likely caused by the simultaneous correction of severe hypokalemia and it might have been prevented by a more cautious and thoughtful management of the patient. This case may very well be considered as an appropriate example to reinforce warning among physicians about the care that should be taken in a clinical setting, in order to prevent rapid changes in serum osmolarity, which may lead to dramatic and sometimes irreversible consequences.

## Consent

Written informed consent was obtained by the patient for the publication of this case report and any accompanying images.

## Abbreviations

ODS: Osmotic demyelination syndrome; [Na+]: Serum sodium concentration; MRI: Magnetic resonance imaging; CPM: Central pontine myelinolysis; EPM: Extra-pontine myelinolysis.

## Competing interests

AP is on the Otsuka Pharmaceutical advisory board for tolvaptan and has received honoraria from Otsuka Pharmaceutical for speaking at symposia; GC, LS, CG, AS declare that they have no competing interests.

## Authors’ contributions

*Acquisition of medical report of the patient*: GC, AS. *Analysis and interpretation of data*: GC, AP, AS. *Drafting of the manuscript*: AP, GC, CG *MRI. examination*: LS. All authors read and approved the final manuscript.

## Pre-publication history

The pre-publication history for this paper can be accessed here:

http://www.biomedcentral.com/1472-6823/14/34/prepub
